# Effects of a whole food diet on immune function and inflammatory phenotype in healthy dogs: A randomized, open-labeled, cross-over clinical trial

**DOI:** 10.3389/fvets.2022.898056

**Published:** 2022-08-23

**Authors:** Jared A. Jaffey, Dan Su, Ross Monasky, Brenna Hanratty, Elizabeth Flannery, Melissa Horman

**Affiliations:** ^1^Department of Specialty Medicine, Midwestern University College of Veterinary Medicine, Glendale, AZ, United States; ^2^JustFoodForDogs LLC, Irvine, CA, United States

**Keywords:** nutrition, inflammation, acute phase proteins, canine, flow cytometry—methods, cytokine

## Abstract

Whole foods in humans decrease inflammation and risk for various diseases, as well as increase weight loss and immune function. Nutrition has been shown to be an integral component in the management of various diseases in dogs but the immunologic and anti-inflammatory effects of whole food diets have not been explored. Therefore, our objective was to assess the effect of feeding a whole food diet on immune function and inflammatory phenotype in healthy dogs. A prospective, randomized, open-labeled, cross-over clinical trial was performed. Sixteen healthy client-owned dogs were fed either a whole food or an extruded dry diet, and after 67 days, they were fed the alternate diet for an additional 67 days. Blood samples were obtained at the completion of each treatment arm (i.e., days 67 and 134). Serum c-reactive protein (CRP), haptoglobin (Hp), and serum amyloid-A (SAA) were measured with ELISA assays. Whole blood cultures were performed with exposure to a phosphate-buffered solution (PBS), lipopolysaccharide (LPS), and lipoteichoic acid (LTA). A canine specific multiplex bead-based assay was then used to measure tumor necrosis factor (TNF)-α, interleukin (IL)-6, IL-10, granulocyte-macrophage colony-stimulating factor (GM-CSF), IL-2, IL-8, and monocyte chemoattractant protein (MCP)-1 concentrations. Granulocyte/monocyte (GM) phagocytosis and oxidative burst associated with *Escherichia coli* were evaluated *via* flow cytometry. Dogs fed a whole food diet had significantly lower TNF-α-to-IL-10 ratios (*P* = 0.05) and higher production of IL-8 (*P* = 0.03) with LTA-exposed leukocytes compared to dogs fed an extruded dry diet. There were no between-treatment differences in the remaining leukocyte cytokine responses, serum CRP, Hp, SAA concentrations, or GM phagocytic and oxidative burst capacities. Whole food diets could have immunomodulatory effects in dogs. Future studies in non-healthy dogs are warranted.

## Introduction

An increasing number of pet owners are seeking alternative pet food options to the standard commercially extruded dry diets, and cooked whole food pet diets have emerged as a popular alternative (1). Formal surveys investigating reasons for this shift in preference have not been published in dogs; however, anecdotal feedback from pet owners in clinical practice suggest that diet composition (e.g., preservatives, additives, unknown ingredients, and contaminants) and a general distrust of large pet food companies are common motivators.

Whole foods in humans decrease inflammation and risk for various diseases as well as increase weight loss and immune function (2–9). Phytonutrients that exist in whole fruits, vegetables, and grains have been identified as the bioactive compounds responsible for some of these benefits (2, 3). Moreover, carotenoids, such as β-carotene, and flavonoids activate and support the immune system (5, 10–12). Whether cooked whole food pet diets provide health benefits compared to conventional extruded dry diets in dogs remains largely unknown (13).

To begin to understand the immunologic effects of whole food diets in dogs, our study aimed to compare granulocyte/monocyte (GM) phagocytic and oxidative burst capacities associated with *Escherichia coli* (*E. coli*), stimulated leukocyte cytokine production, and serum inflammatory biomarkers in healthy dogs fed either a whole food or a commercially available extruded dry diet. We hypothesized that feeding a whole food diet would improve immune function while concomitantly decreasing inflammation compared to commercially available extruded dry diets.

## Materials and methods

### Animals

This was a prospective, randomized, open-labeled, cross-over, clinical trial. Healthy dogs between the ages of 1 and 10 years, of any breed or sex, fed a commercially available high-temperature extruded dry diet were eligible for enrollment. The study protocol was approved by the Midwestern University Animal Care and Use Committee (protocol #2992) with written owner consent. Dogs were considered healthy based on history, physical examination, and after review of hematology, serum chemistry and urinalysis results by a single board-certified small animal internist (JAJ). Dogs could not have a body condition score (BCS) of > 6/9, had an illness, or been administered medications, except monthly parasiticides, been vaccinated, or had a diet change within 60 days of enrollment. Exclusion criteria were refusal to eat the whole food diet, administration of any drug that could affect immune function or inflammatory phenotype, and if both treatment arms of the study were not completed.

### Whole food and extruded dry diets

The whole food diet was a commercially available diet (JustFoodForDogs LLC) formulated by a board-certified veterinary nutritionist that used fully cooked, human-grade ingredients, and is formulated to meet canine growth nutrient levels established by the Association of American Feed Control Officials (AAFCO). The primary ingredients in the whole food diet included chicken thigh, long grain white rice, spinach, carrots, apples, chicken gizzard, chicken liver, and fish oil. The nutrient profile of the whole food diet can be found in Table 1. A board-certified veterinary nutritionist reviewed the owner reported diet history of the dogs to establish the initial feeding amount of the whole food diet. The dogs were weighed 2–3 weeks after starting the whole food diet to either maintain or adjust feeding amounts to prevent weight gain or loss. The extruded dry diet type and amount fed to each dog was the same maintenance diet and feeding regimen used before enrollment in the study. Details regarding the types of extruded dry diets used in this study can be found in Supplemental material.

**Table 1:** Nutrient analysis of the whole food diet.

**Nutrients**	**Nutrient content per 1,000 kcal ME**
Crude protein (g)	72
Crude fat (g)	26
Crude fiber (g)	1.4
Carbohydrate (by difference) (g)	100
EPA and DHA (g)	0.17
Calcium (g)	3.4
Phosphorus (g)	2.7
Vitamin D (IU)	144
Vitamin E (IU)	15
Vitamin A (IU)	8,287
Zinc (mg)	39
Omega 6:3 ratio	10:1
ME (calc), kcal/kg	1,535

EPA, eicosapentaenoic acid; DHA, docosahexaenoic acid; g, grams; mg, milligrams; IU, international unit; ME, metabolizable energy; kcal, kilocalorie; kg, kilogram.

### Study design

**Figure 1:**
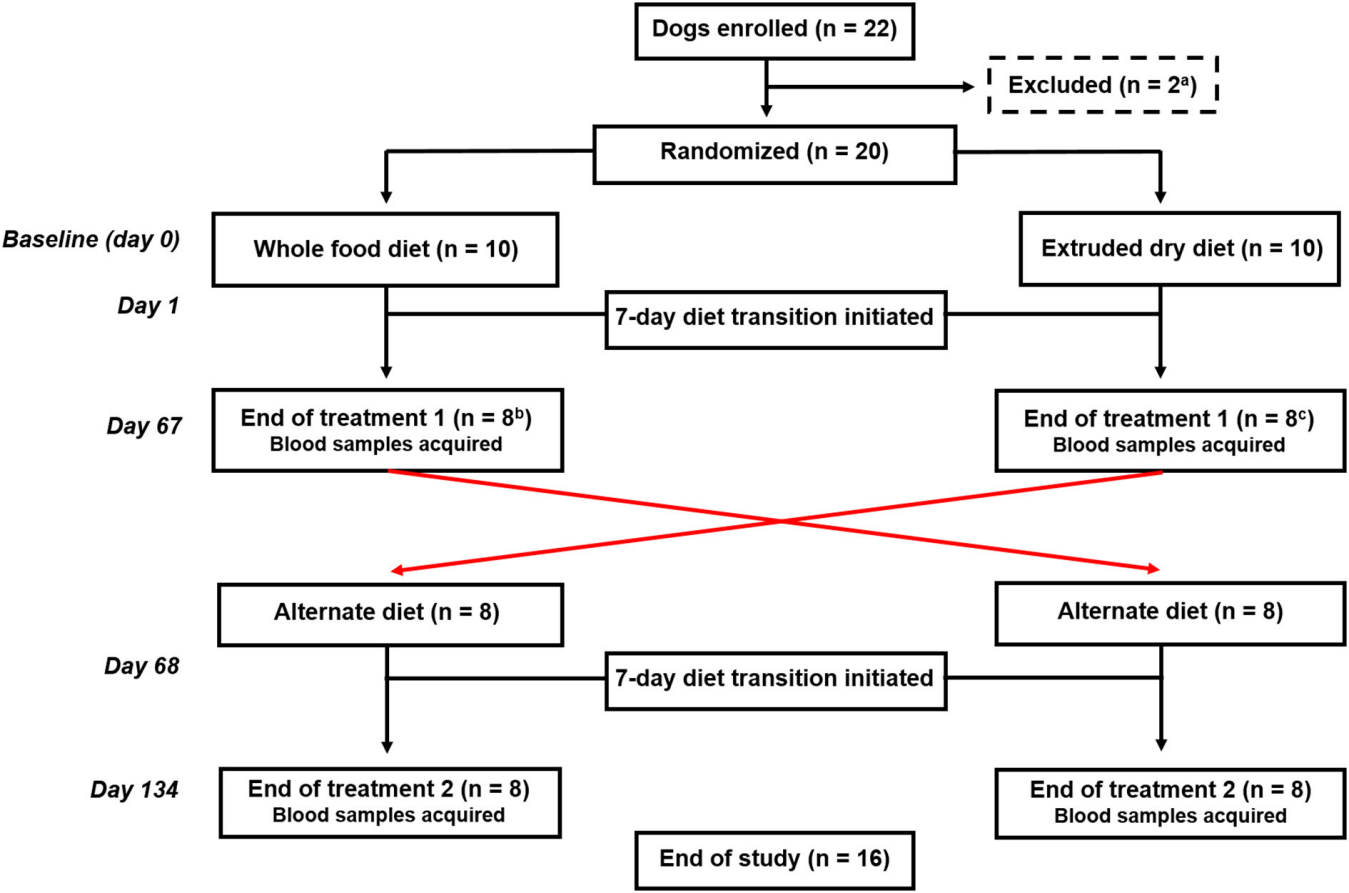
Flowchart documenting the number of dogs recruited to each treatment and their progress through the trial. ^a^Two dogs were excluded before randomization because of being relocated outside of the geographic region. ^b^Two dogs fed the whole food diet were excluded before completion of the first treatment arm because of one each of refusal to eat the food and another dog was withdrawn by the owner because vomiting and diarrhea that developed during the diet transition period. ^c^Two dogs fed an extruded dry diet were excluded because of the administration of a prohibited medication before the completion of the first treatment arm.

Dogs were randomized using a research randomizer (https://www.random.org) to be fed either a whole food or an extruded dry diet, and after 67 days, they were fed the alternate diet for an additional 67 days (Figure 1). Each treatment period included a 7-day diet transition phase outlined as: days 1–2 (75% kcal from previous diet + 25% kcal from new diet), days 3–4 (50% kcal from previous diet + 50% kcal from new diet), days 5–6 (25% kcal from previous diet + 75% kcal from new diet), and day 7 (100% kcal from new diet). Blood samples were obtained at the end of each treatment arm (i.e., days 67 and 134).

### Blood sample collection and processing

Blood samples were collected and processed within 1 h of collection. Blood samples for c-reactive protein (CRP), serum amyloid-A (SAA), and haptoglobin (Hp) measurement were collected into serum separator tubes, centrifuged, and serum placed in freezer-resistant conical microcentrifuge tubes and stored at −80°C for batch analysis. Lithium heparinized blood was used for immune function testing.

### C-reactive protein, serum amyloid-A, and haptoglobin

Serum samples were thawed and analyzed in duplicate for CRP, SAA, and HP. Serum Hp and SAA concentrations were measured with commercially available colorimetric methods that were validated previously in dogs (Tridelta Phase Haptoglobin Assay, Tridelta Ltd, Maynooth, Ireland.) (14). Serum CRP was measured with a commercially available canine-specific sandwich ELISA (Abcam) as previously described (15). Manufacturer provided lower limits of detection were: CRP (1.1 ng/mL), Hp (0.078 g/L), and SAA (0.005 μg/mL).

### Leukocyte cytokine production

Whole blood from tubes containing lithium heparin were diluted 1:2 with RPMI 1,640 culture medium containing 200 U of penicillin/mL and 200 mg of streptomycin/mL, transferred to 24-well plates, and stimulated with lipoteichoic acid (LTA) from *Streptococcus faecalis* (final concentration 1 μg/mL, Sigma Aldrich), lipopolysaccharide (LPS) from *E. coli* 0127:B8 (final concentration, 100 ng/mL, Sigma Aldrich), or phosphate-buffered solution (PBS) as a negative control. Samples were incubated in the dark at 37°C in 5% CO_2_ for 24 h. The samples were then centrifuged (400 g for 7 min) and supernatant collected and stored at −80°C for batch analysis. For analysis, samples were thawed, and then tumor necrosis factor (TNF)-α, interleukin (IL)-6, IL-10, granulocyte-macrophage colony-stimulating factor (GM-CSF), IL-2, IL-8, and monocyte chemoattractant protein (MCP)-1 were quantified with a canine cytokine-specific multiplex bead-based assay (Milliplex MAP, EMD Millipore Corp.) as described elsewhere (16). The median fluorescence intensity and cytokine concentration in each sample was measured in duplicate with appropriate controls and associated data analysis software (Milliplex Analyst version 5.1, EMD Millipore Corp.). The lower limit of detection for TNF-α, IL-6, IL-10, GM-CSF, IL-2, and MCP-1 was 48.8 ρg/mL. The lower limit of detection for IL-8 was 195.3 ρg/mL.

### Oxidative burst induced by *E. coli*

Oxidative burst capacity in GM was determined with a commercially available assay (PhagoBurst kit, Orpegen Pharma) validated for use in canines. Heparinized blood from each dog was incubated with 20 μL of opsonized-*E. coli* strain LE392 or control solution for 10 min in a 37°C water bath (17). Samples were then incubated for 10 min at 37°C in a water bath with 20 μL of dihydrorhodamine-123, a fluorogenic substrate, which is oxidized to rhodamine 123 by oxygen intermediates. After cessation of this reaction, erythrocytes were lysed, the cells were washed, and 200 μL of DNA staining solution (R-phycoerythrin) was added to facilitate exclusion of aggregated artifacts of bacteria or cells that do not have intact DNA to bind.

### Phagocytosis of *E. coli*

Phagocytic capacity of GM was determined with a commercially available assay (PhagoTest, Orpegen Pharma), validated for use in canines. Heparinized blood was incubated with FITC-labeled, opsonized-*E. coli* strain LE392. The control samples were incubated on ice for 10 min while test samples were incubated in a 37°C water bath for 10 min (18). Phagocytosis was then arrested with samples being placed on ice and quenching solution was added to extinguish surface bound FITC-labeled-*E. coli*. The cells were then washed, erythrocytes lysed, and cells washed again before DNA staining (R-phycoerythrin) solution was added to facilitate exclusion of aggregated artifacts of bacteria or cells without intact DNA.

### Flow cytometry

Flow cytometry was performed at the Midwestern University College of Veterinary Medicine Immunology Laboratory using a flow cytometer (Guava easyCyte HT, Luminex Corporation, Texas, United States) and associated data analysis software (GuavaSoft 3.2, Luminex Corporation). A minimum of 20,000 events/sample were recorded. The gating scheme is outlined in Supplementary Figure 1. For assessment of phagocytosis, data was recorded as the percentage of GM cells having internalized FITC-labeled *E. coli*, as well as their mean fluorescent intensity (MFI), a method of quantifying the phagocytosed bacteria per cell. Data for assessment of oxidative burst was recorded as the percentage of GM cells containing rhodamine 123 as a measure of oxidative burst, and the MFI, the relative robustness of oxidative burst reaction produced per cell.

### Adverse events

Short-term adverse events that occurred throughout the study period were recorded. An adverse event was defined as any observation, unwanted experience, or reaction that was unfavorable, unintended, and occurred after initiation of the study (day 1).

### Statistical analysis

Statistical analyses were performed using a commercial software (SigmaPlot, SyStat Software Inc., California, United States). Normality was determined using the Shapiro-Wilk test. Normally distributed data were presented as mean and standard deviation (SD), while non-normally distributed data were presented as median and interquartile range (IQR). Categorical data were presented as proportions. Wilcoxon's signed-rank tests were used for between-treatment comparisons of serum CRP, Hp, and SAA concentrations as well as markers of GM phagocytic and oxidative burst capacities. Likewise, between-treatment comparisons of leukocyte cytokine production based on individual stimulant type (i.e., PBS, LPS, and LTA) were made with Wilcoxon's signed-rank tests. Between-stimulant comparisons of leukocyte cytokine production, irrespective of treatment (i.e., whole food diet or extruded dry diet) were made using Kruskal-Wallis analysis of variance on ranks with *post-hoc* Tukey tests for multiple comparisons. Data are graphically illustrated using box and whisker plots with outliers defined using the Tukey method in which outliers are values 1.5 × IQR below or above the 25th and 75th quartiles, respectively. Outliers were not excluded from statistical analyses. When the measured cytokine, CRP, SAA, and Hp concentration fell below the lower limit of detection for the respective assay, data were recorded at the lower limit of detection for statistical purposes. A *P*-value of ≤ 0.05 was considered significant.

## Results

### Animal cohort

Twenty-two dogs were enrolled (Figure 1). Two dogs were excluded before randomization because of being relocated out of the geographic region. The remaining dogs were randomized to be fed either the whole food diet (*n* = 10) or an extruded dry diet (*n* = 10). Four dogs were subsequently excluded before the end of the first treatment arm. One dog refused to eat the whole food diet and another dog was withdrawn by the owner because of vomiting and diarrhea that developed during the diet transition period to the whole food diet. The remaining two dogs were excluded because they were administered Cytopoint® for newly diagnosed atopic dermatitis. Sixteen dogs completed both treatment arms and represented the final cohort.

A total of 11 mixed breed dogs and 5 purebred dogs were included. Purebred dogs included one each of Chihuahua, Staffordshire terrier, Labrador retriever, Australian shepherd, and Siberian husky. The mean age and weight were 3.1 years (SD, 1.6) and 22.3 kgs (SD, 8.5), respectively. Eighty one percent (13/16) of dogs had a BCS of 5/9 and the remaining 19% (3/16) had a BCS of 6/9. Four dogs had caloric adjustments one time after transition to the whole food diet. The adjustment in all dogs was a reduction in calories (median, 10%; range, 5–20%).

Fifty percent (8/16) of dogs experienced at least one adverse event during the study period. All adverse events occurred during the transition period to the whole food diet. Adverse events included transient vomiting, diarrhea, or both, that resolved without intervention (*n* = 6). One dog had decreased frequency of defecation. The remaining dog had vomiting, diarrhea, and hyporexia that resolved with the administration of metronidazole, maropitant, and omeprazole for 7 days.

### C-reactive protein, haptoglobin, and serum amyloid-A

There was no difference in serum CRP (*P* = 0.14), Hp (*P* = 0.42), or SAA (*P* = 0.78) concentrations in dogs fed the whole food diet compared to an extruded dry diet (Table 2).

**Table 2:** Comparison of serum c-reactive protein, haptoglobin, and serum amyloid-A concentrations as well as the granulocyte/monocyte phagocytic and oxidative burst capacities in dogs fed a whole food diet or extruded dry diet in a cross-over study design.

**Variable**	**Whole food diet** **(*n* = 16)**	**Extruded dry diet** **(*n* = 16)**	***P*-value**
C-reactive protein (ng/mL)	46.4 (45.7)	42.0 (39)	0.14
Haptoglobin (g/L)	0.25 (0.67)	0.24 (0.42)	0.42
Serum amyloid-A (μg/mL)	0.87 (0.70)	0.83 (0.30)	0.78
Phagocytosis (%)	51.5 (29.8)	60.9 (36.4)	0.11
Phagocytosis (MFI)	6,475 (1,979.4)	5,613 (2,126)	0.82
Oxidative burst (%)	60.2 (23.6)	62 (11.5)	0.90
Oxidative burst (MFI)	1,527.1 (792.2)	1,286.9 (840.5)	0.82

Data presented as median (interquartile range).

MFI, mean fluorescent intensity.

### Leukocyte cytokine responses

There were significant between-stimulant differences in leukocyte production of TNF-α, IL-6, IL-10, GM-CSF, IL-8, and MCP-1 (*P* < 0.001), but not IL-2 (*P* = 0.96), irrespective of treatment (i.e., whole food diet or extruded dry diet) (Table 3). Leukocytes stimulated with LPS produced significantly greater TNF-α, IL-6, IL-10, GM-CSF, IL-8, and MCP-1 than leukocytes exposed to PBS (*P* < 0.001; Table 3). Similarly, LPS-stimulated leukocytes produced significantly greater TNF-α, IL-6, IL-10, GM-CSF, and IL-8 than LTA-exposed leukocytes (*P* < 0.001; Table 3). Lastly, TNF-α (*P* = 0.001) and MCP-1 (*P* < 0.001) were significantly higher with LTA-stimulated leukocytes compared to PBS-exposed leukocytes (Table 3).

**Table 3:** Comparison of leukocyte cytokine production based on stimulant, irrespective of treatment (i.e., whole food diet or extruded dry diet).

**Cytokine (ρg/mL)**	**PBS**	**LPS**	**LTA**	***P*** **-value**		
				**a**	**b**	**c**
TNF-α	48.8 (0)	1,204.5 (973.3)	75.6 (86.8)	<0.001	<0.001	0.001
IL-6	48.8 (0)	275.6 (196.4)	48.8 (29.2)	<0.001	<0.001	0.26
IL-10	48.8 (0)	845.7 (691.2)	48.8 (19.6)	<0.001	<0.001	0.32
GM-CSF	48.8 (0)	382.4 (263.7)	48.8 (0)	<0.001	<0.001	0.99
IL-2	48.8 (0)	48.8 (0)	48.8 (0)	0.96		
IL-8	195.3 (0)	8,454.5 (3,523.3)	242.7 (727)	<0.001	<0.001	0.07
MCP-1	1,005.7 (465.4)	2,935.5 (2,780)	2,402.5 (1,826.8)	<0.001	0.36	<0.001

Data presented as median (interquartile range).

PBS, phosphate-buffered solution; LPS, lipopolysaccharide; LTA, lipoteichoic acid; TNF, tumor necrosis factor; IL, interleukin; GM-CSF, granulocyte-macrophage colony-stimulating factor; MCP, monocyte chemoattractant protein.

^a^LPS vs. PBS.

^b^LPS vs. LTA.

^c^LTA vs. PBS.

**Figure 2:**
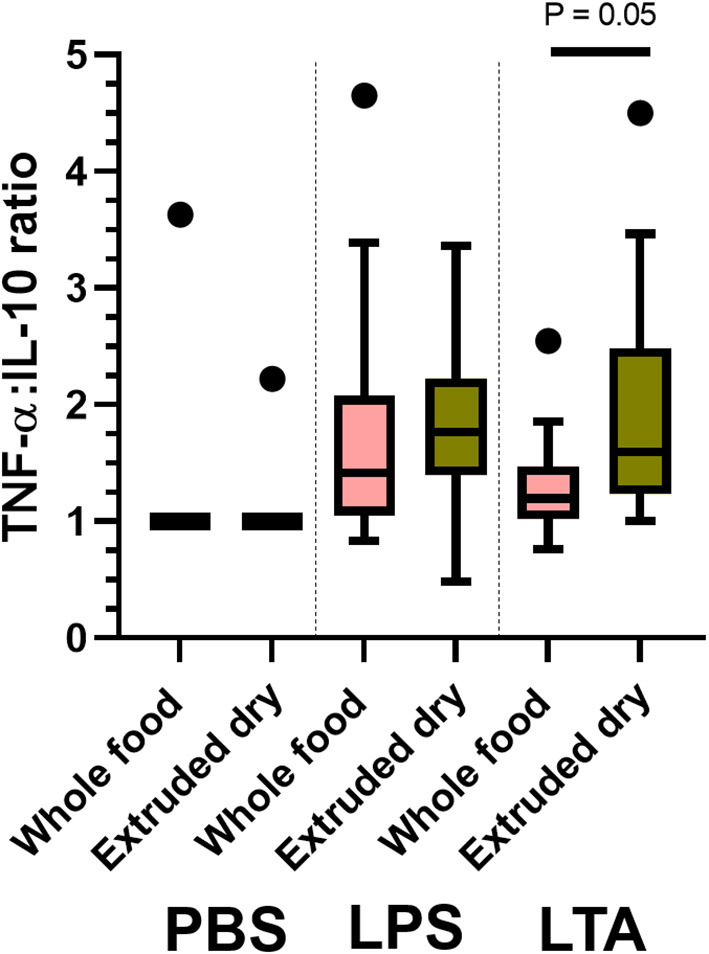
Box and whisker plots comparing between-treatment tumor necrosis factor (TNF)-α-to-interleukin (IL)-10 ratios with leukocytes exposed to phosphate-buffered solution (PBS); lipopolysaccharide (LPS); and lipoteichoic acid (LTA). The top and bottom of the boxes represent the 75th and 25th quartiles, respectively, with the black horizontal line representing the median. The whiskers extend up to 1.5 × interquartile range below and above the 25th and 75th quartiles, respectively. Closed circles above the whiskers represent outlier values.

Next, we compared leukocyte cytokine production between treatments based on individual stimulant type (i.e., PBS, LPS, and LTA). Leukocytes from dogs fed the whole food diet produced significantly higher IL-8 than dogs fed an extruded dry diet when stimulated with LTA (*P* = 0.03; Table 4). There were no other between-treatment differences of individual leukocyte cytokine production (Table 4). The TNF-α-to-IL-10 ratio was significantly lower in dogs fed the whole food diet compared to dogs fed an extruded dry diet when leukocytes were stimulated with LTA (*P* = 0.05), but not with PBS or LPS exposure (Figure 2).

**Table 4:** Comparison of leukocyte cytokine production based on stimulant in dogs fed a whole food diet or extruded dry diet in a cross-over study design.

**Cytokine (ρg/mL)**	**Whole food diet** **(*n* = 16)**	**Extruded dry diet** **(*n* = 16)**	***P*-value**
**PBS**			
TNF-α	48.8 (0)	48.8 (0)	1.00
IL-6	48.8 (0)	48.8 (0)	1.00
IL-10	48.8 (0)	48.8 (0)	1.00
GM-CSF	48.8 (0)	48.8 (0)	1.00
IL-2	48.8 (0)	48.8 (0)	0.25
IL-8	195.3 (0.4)	195.3 (0)	0.44
MCP-1	995.1 (368.8)	1,064.2 (692.4)	0.86
**LPS**			
TNF-α	1,204.5 (896.3)	1,222.5 (1,027.3)	0.94
IL-6	275.6 (187.4)	274.2 (199.9)	0.50
IL-10	838.6 (692.7)	845.7 (445.8)	0.40
GM-CSF	325.9 (247.2)	390 (436.5)	0.09
IL-2	48.8 (0)	48.8 (0)	0.75
IL-8	8,250 (3,466.8)	8,556 (5,088)	0.32
MCP-1	2,266 (1,836.8)	3,400.5 (2,949.3)	0.71
**LTA**			
TNF-α	66 (66.3)	88.5 (103.2)	0.45
IL-6	48.8 (44.7)	48.8 (19.8)	0.08
IL-10	48.8 (49.1)	48.8 (0)	0.20
GM-CSF	48.8 (0)	48.8 (0)	1.00
IL-2	48.8 (0)	48.8 (0)	0.75
IL-8	393.9 (1,345)	222.9 (249)	0.03
MCP-1	2,419 (1,534)	2,402.5 (1,954)	0.53

Data presented as median (interquartile range).

PBS, phosphate-buffered solution; LPS, lipopolysaccharide; LTA, lipoteichoic acid; TNF, tumor necrosis factor; IL, interleukin; GM-CSF, granulocyte-macrophage colony-stimulating factor; MCP, monocyte chemoattractant protein.

### Phagocytosis and oxidative burst

There was no significant difference in the percentage of GM that phagocytized opsonized-*E. coli* (*P* = 0.11), or number of phagocytized opsonized-*E. coli* per cell (*P* = 0.82), between dogs fed the whole food diet or an extruded dry diet (Table 1). Likewise, a difference in the percentage of GM that had performed *E. coli*-induced oxidative burst (*P* = 0.90), as well as the *E. coli*-induced oxidative burst intensity per cell (*P* = 0.82) was not identified (Table 2).

## Discussion

This is the first clinical trial to investigate the effects of feeding a whole food diet on immune function and inflammatory phenotype in dogs. In our investigation, we found that dogs fed a whole food diet had significantly lower TNF-α-to-IL-10 ratios and higher production of IL-8 with LTA-exposed leukocytes compared to dogs fed an extruded dry diet. There were no differences in the remaining leukocyte cytokine responses, serum CRP, Hp, and SAA concentrations, or GM phagocytic and oxidative burst capacities.

Dogs fed the whole food diet had lower TNF-α-to-IL-10 ratios with leukocyte exposure to LTA. Tumor necrosis factor-α-IL-10 ratios are used to investigate shifts in host inflammatory phenotype with decreases in this ratio correlating with a decrease in inflammation (i.e., anti-inflammatory shift) (19). Our results indicate that whole food diet could have anti-inflammatory effects in dogs with disorders causing systemic inflammation. This theory is based on our finding of a significant reduction in TNF-α-to-IL-10 ratio under *in vitro* simulated conditions of inflammation with stimulation of leukocytes. These results corroborate findings in humans that whole food diet composition can reduce biomarkers of systemic inflammation including CRP and pro-inflammatory cytokines (e.g., TNF-α, IL-6) (4, 11, 20–23).

The whole food diet used in our study might provide anti-inflammatory effects for several reasons. One potential reason is that the whole food diet presumably contained less advanced glycation end products (AGEs) because it was prepared at a lower cooking temperature than the extruded dry diets (24). These AGEs are absorbed from diets, and have been associated with age-related and chronic inflammatory conditions such as osteoarthritis, atherosclerosis, nephropathy, and diabetes mellitus in humans (25). Intervention studies in humans showed a decrease in inflammation in subjects on a low-AGE diet (26). Many of these age-related diseases are also found in dogs, and elevated levels of AGEs in tissue proteins were observed in aging dogs affected by these conditions (25). The whole food diet also contains fish oil as a source of eicosapentaenoic acid (EPA) and docosahexaenoic acid (DHA), which are known to have less pro-inflammatory effects than omega-6 polyunsaturated fatty acids (27). It is unlikely that the EPA and DHA would be the sole contributing factor to the anti-inflammatory effect of the whole food diet because fish oil is a commonly used ingredient in extruded dry dog foods. Other nutrients such as vitamin D and zinc might also play a role in the anti-inflammatory effect of the whole food diet (17, 18, 28, 29). Comparisons of individual components of the whole food diet and extruded dry diet could not be performed because this information was inconsistently available among the extruded dry diets. Additional investigation is needed to determine which components of the whole food diet were responsible for our findings.

Leukocyte production of IL-8 was higher with LTA-exposed leukocytes in dogs fed the whole food diet compared to an extruded dry diet. Interleukin-8 is secreted by monocytes, neutrophils, epithelial, fibroblast, endothelial, and mesothelial cells in response to an inflammatory stimulus (30). This chemokine induces chemotaxis of target cells, primarily neutrophils, but also fibroblasts, causing them to migrate toward sites of infection (30–32). Interleukin-8 also increases neutrophil phagocytosis and oxidative burst, which increases pathogen killing efficiency (32, 33). Collectively, IL-8 plays an important role in clearance of infections and wound healing. Taken together, our results suggest that by increasing leukocyte production of IL-8 under *in vitro* simulated conditions of inflammation, the whole food diet could improve the host innate immune response to infections. It must also be considered that uncontrolled overexpression of IL-8 can lead to amplified inflammation in chronic inflammatory diseases (34). However, exaggerated chronic overexpression of IL-8 was an unlikely sequela to feeding a whole food diet in our study because of the lack of between-treatment difference in IL-8 concentrations with non-stimulated PBS-exposed leukocytes. In addition, overexpression of IL-8 would be expected to coincide with a concomitant increase in pro-inflammatory biomarkers, which were not identified. Additional studies investigating the effect of whole food diet on leukocyte IL-8 production in circulation and its clinical relevance in dogs are warranted.

There were no differences in serum inflammatory biomarkers including CRP, Hp, SAA, nor were there differences in phagocytic or oxidative burst capacities associated with *E. coli* in dogs fed the whole food diet compared to an extruded dry diet. Healthy dogs were included in this study making it difficult to ascertain if the whole food diet decreased inflammation or enhanced phagocytosis and oxidative burst because these dogs presumably lacked a pro-inflammatory milieu or immune dysregulation at baseline. Future clinical trials in dogs with disorders associated with chronic systemic inflammation are needed to further investigate the immunomodulatory effects of whole food diets.

This study had several limitations that require further elucidation. The dogs in this study were not fed a uniform control extruded dry diet. The use of a uniform extruded dry diet would have allowed for more controlled comparisons with less variability from heterogeneous diet formulations; however, these results would not have been as applicable to the general population of dogs that consume a variety of extruded dry diets. Moreover, the use of two novel diets in this study (i.e., whole food and uniform extruded dry diet) could have increased the incidence of adverse events and subsequently contributed to the withdrawal of dogs by their owners. Half of the dogs in this study experienced at least one adverse event during the transition period from the extruded dry diet to the whole food diet. The clinical signs characterized as adverse events in this study (i.e., vomiting, diarrhea, and hyporexia), are relatively common in dogs during the transition to a new diet. These adverse events were mild, transient, and resolved without intervention in most cases within 7 days, and would not have been expected to affect immunologic testing performed ~60 days later. Next, our study examined the effects of a single type of whole food diet, and thus the results cannot be extrapolated to other similarly prepared products due to various potential inherent differences. The whole food diet utilized in this study is a product that has been approved for canine maintenance *via* an AAFCO feeding trial, and is formulated to meet the requirements for puppy growth. While other whole food diets might be similarly processed, their nutrient profile could differ from the diet used in this study, leading to different effects on immune function and inflammation. This study investigated the effects of feeding a whole food diet on the systemic inflammatory phenotype in blood samples, which does not necessarily predict what happens in the intestinal mucosa. Future studies could consider evaluating the impact that feeding a whole food diet has on the immunologic microenvironment of intestinal mucosal cells. There is growing evidence in humans that the intestinal microbiome can indirectly influence systemic host immune responses (35). Therefore, it is possible the whole food diet caused a shift in the intestinal microbiota that, in turn, altered systemic immune responses. Consideration should be given to the possible effect that whole food diets have on the intestinal microbiota and the indirect effect on immune responsiveness in future studies. Lastly, this was an exploratory study with a conservative sample population, which could have impeded the identification of significant differences (i.e., type II error).

## Conclusion

In conclusion, our results indicate that feeding dogs a whole food diet could have immunomodulatory effects. Future studies comprised of non-healthy dogs are needed to further explore the anti-inflammatory and immunologic effects of whole food diets.

## Data availability statement

The raw data supporting the conclusions of this article will be made available by the authors, without undue reservation.

## Ethics statement

The animal study was reviewed and approved by the Midwestern University Animal Care and Use Committee (protocol #2992) with written owner consent. Written informed consent was obtained from the owners for the participation of their animals in this study.

## Author contributions

JJ: study design, data acquisition, analysis and interpretation of data, and drafted manuscript. DS: study design, analysis and interpretation of data, and drafted manuscript. RM: data acquisition, analysis of data, and drafted manuscript. BH, EF, and MH: study recruitment, data acquisition, and drafted manuscript. All authors contributed to the article and approved the submitted version.

## Funding

The authors declare that this study received funding from JustFoodForDogs LLC. DS was employed by the funder and had the following involvement in the study: study design, analysis and interpretation of data, and drafting of manuscript.

## Conflict of interest

DS was employed by JustFoodForDogs LLC. The remaining authors declare that the research was conducted in the absence of any commercial or financial relationships that could be construed as a potential conflict of interest.

## Publisher's note

All claims expressed in this article are solely those of the authors and do not necessarily represent those of their affiliated organizations, or those of the publisher, the editors and the reviewers. Any product that may be evaluated in this article, or claim that may be made by its manufacturer, is not guaranteed or endorsed by the publisher.

## Supplementary material

Supplementary Figure 1 Gating scheme for flow cytometry of phagocytosis and oxidative burst. Granulocytes and monocytes were gated on a forward vs. side scatter plot **(A)**. Next, R-phycoerythrin (PE)-labeled DNA stain was used to exclude aggregates of bacteria or dead cells, with positive-staining cells identified and gated **(B)**. These gated cells were then applied to a histogram to determine percentage of FITC-positive cells and their mean fluorescent intensity. Representative histograms of a negative control **(C)** and positive sample **(D)** are shown.
