# Supplementary figures and images for: Effects of a whole food diet on immune function and inflammatory phenotype in healthy dogs: A randomized, open-labeled, cross-over clinical trial
Source: Front Vet Sci. 2022 Aug 23;9:898056. doi: 10.3389/fvets.2022.898056 (PMC9447376; doi:10.3389/fvets.2022.898056)

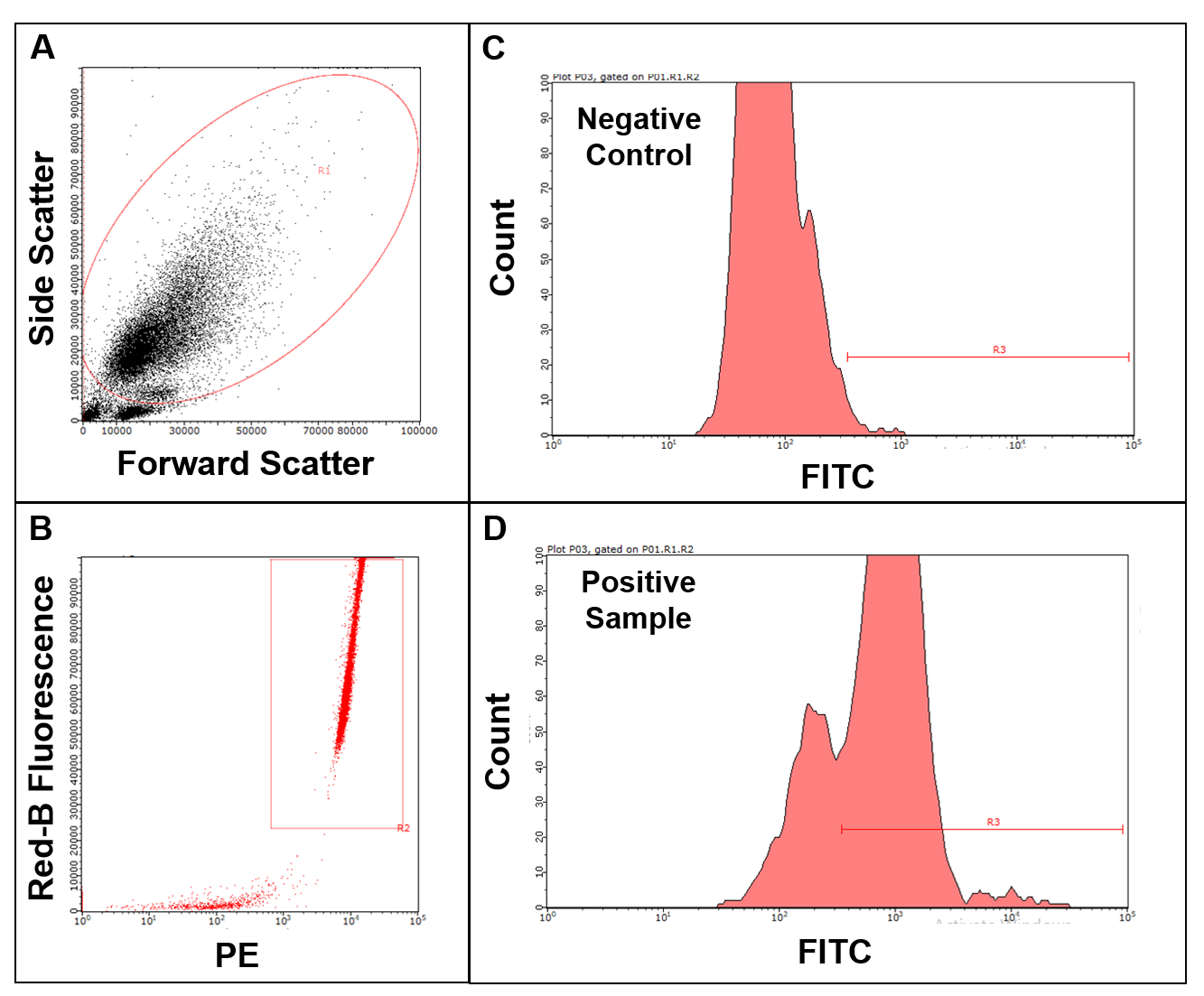

Supplement: Supplementary Figure 1 — Gating scheme for flow cytometry of phagocytosis and oxidative burst. Granulocytes and monocytes were gated on a forward vs. side scatter plot (A). Next, R-phycoerythrin (PE)-labeled DNA stain was used to exclude aggregates of bacteria or dead cells, with positive-staining cells identified and gated (B). These gated cells were then applied to a histogram to determine percentage of FITC-positive cells and their mean fluorescent intensity. Representative histograms of a negative control (C) and positive sample (D) are shown. [file Image_1.TIF]
