# Supplementary material for: Effects of a whole food diet on immune function and inflammatory phenotype in healthy dogs: A randomized, open-labeled, cross-over clinical trial
Source: Front Vet Sci. 2022 Aug 23;9:898056. doi: 10.3389/fvets.2022.898056 (PMC9447376; doi:10.3389/fvets.2022.898056)
Supplement: Supplementary file 2 [file Data_Sheet_1.docx]

**Supplemental Material**

The distribution of extruded dry diets used in this study included: Purina Pro Plan Large Breed Chicken & Rice Formula (3) [Nestle Purina PetCare Company, USA], Kirkland Signature Adult Formula Lamb, Rice & Vegetable (3) [Diamond Pet Foods Inc., USA], Purina ONE SmartBlend Chicken & Rice Adult Formula (2), American Journey Active Life Formula Lamb Brown Rice & Vegetables [PetsMart, Chewy, USA], Hill’s Science Diet Adult Chicken and Barley [Hill’s Pet Nutrition Inc., USA], Taste of the Wild High Prairie [Diamond Pet Foods Inc., USA], Purina Pro Plan Savor Adult Shredded Blend Salmon & Rice, Purina Pro Plan Focus Chicken & Rice Formula Dry Puppy Food, Hill’s Science Diet Adult Chicken & Barley Recipe, Purina Pro Plan Savor Shredded Blend Lamb Rice Adult Dog Food, Hill’s Science Diet Adult Small Bites Chicken & Barley.
